# Linking Polygenic Risk of Schizophrenia to Variation in Magnetic Resonance Imaging Brain Measures: A Comprehensive Systematic Review

**DOI:** 10.1093/schbul/sbad087

**Published:** 2023-06-24

**Authors:** Hadis Jameei, Divyangana Rakesh, Andrew Zalesky, Murray J Cairns, William R Reay, Naomi R Wray, Maria A Di Biase

**Affiliations:** Melbourne Neuropsychiatry Centre, Department of Psychiatry, The University of Melbourne and Melbourne Health, Carlton South, VIC, Australia; Melbourne Neuropsychiatry Centre, Department of Psychiatry, The University of Melbourne and Melbourne Health, Carlton South, VIC, Australia; Melbourne Neuropsychiatry Centre, Department of Psychiatry, The University of Melbourne and Melbourne Health, Carlton South, VIC, Australia; Faculty of Engineering and Information Technology, The University of Melbourne, Parkville, VIC, Australia; School of Biomedical Sciences and Pharmacy, The University of Newcastle, Newcastle, NSW, Australia; Precision Medicine Research Program, Hunter Medical Research Institute, Newcastle, NSW, Australia; School of Biomedical Sciences and Pharmacy, The University of Newcastle, Newcastle, NSW, Australia; Precision Medicine Research Program, Hunter Medical Research Institute, Newcastle, NSW, Australia; Institute for Molecular Bioscience, The University of Queensland, Brisbane, QLD, Australia; Queensland Brain Institute, The University of Queensland, Brisbane, QLD, Australia; Melbourne Neuropsychiatry Centre, Department of Psychiatry, The University of Melbourne and Melbourne Health, Carlton South, VIC, Australia; Department of Anatomy and Physiology, School of Biomedical Sciences, The University of Melbourne, VIC, Australia; Department of Psychiatry, Brigham and Women’s Hospital, Harvard Medical School, Boston, MA, USA

**Keywords:** magnetic resonance imaging, single nucleotide polymorphisms, genetic risk, brain structure, brain function, psychosis

## Abstract

**Background and hypothesis:**

Schizophrenia is highly heritable, with a polygenic effect of many genes conferring risk. Evidence on whether cumulative risk also predicts alterations in brain morphology and function is inconsistent. This systematic review examined evidence for schizophrenia polygenic risk score (sczPRS) associations with commonly used magnetic resonance imaging (MRI) measures. We expected consistent evidence to emerge for significant sczPRS associations with variation in structure and function, specifically in frontal, temporal, and insula cortices that are commonly implicated in schizophrenia pathophysiology.

**Study Design:**

In accordance with Preferred Reporting Items for Systematic Reviews and Meta-Analyses (PRISMA) guidelines, we searched MEDLINE, Embase, and PsycINFO for peer-reviewed studies published between January 2013 and March 2022. Studies were screened against predetermined criteria and National Institutes of Health (NIH) quality assessment tools.

**Study Results:**

In total, 57 studies of T1-weighted structural, diffusion, and functional MRI were included (age range = 9–80 years, Nrange = 64–76 644). We observed moderate, albeit preliminary, evidence for higher sczPRS predicting global reductions in cortical thickness and widespread variation in functional connectivity, and to a lesser extent, region-specific reductions in frontal and temporal volume and thickness. Conversely, sczPRS does not predict whole-brain surface area or gray/white matter volume. Limited evidence emerged for sczPRS associations with diffusion tensor measures of white matter microstructure in a large community sample and smaller cohorts of children and young adults. These findings were broadly consistent across community and clinical populations.

**Conclusions:**

Our review supports the hypothesis that schizophrenia is a disorder of disrupted within and between-region brain connectivity, and points to specific whole-brain and regional MRI metrics that may provide useful intermediate phenotypes.

## Introduction

Schizophrenia is a severe neuropsychiatric disorder, characterized by a substantial genetic component. While the neurobiology of schizophrenia remains largely unestablished, scores of magnetic resonance imaging (MRI) studies over the last three decades demonstrate structural and functional brain differences in individuals with schizophrenia compared to healthy controls.^1–3^ Establishing links between these commonly reported brain abnormalities and the underlying genetic architecture of schizophrenia may accelerate progress toward a mechanistic understanding of this highly complex trait.

The most frequently reported MRI findings in schizophrenia include whole-brain and regional (frontal, temporal, and insula) reductions in cortical volume and thickness, as well as widespread disruptions in structural and functional brain connectivity.^4^ Evidence of genetic contributions to these brain alterations stems from three primary sources. First, a recent large-scale analysis in the UK Biobank reported high single nucleotide polymorphism (SNP)-based heritability for volumetric measures (mean of ~40% across brain regions), cortical thickness (mean of ~20% across regions), and resting-state brain function (mean of ~20% across regions and ~5% across connections).^5–7^ Second, brain alterations, including reduced gray matter volume and cortical thickness, as well as alterations in white matter microstructure,^8^ have been observed in healthy co-twins of schizophrenia probands, implying shared genetic variance between schizophrenia and its associated brain imaging alterations.^9^ Third, several previous studies indicate strong genetic overlap between schizophrenia and features of brain morphology and function.^10–12^

The SNP-based heritability estimate for schizophrenia suggests that around a third of the genetic liability to schizophrenia is explained by common variants that each confer small increments of risk.^13^ Genome-wide association studies (GWAS) have identified hundreds of such variant associations with schizophrenia,^14–27^ with the latest GWAS (psychiatric genomics consortium-3) uncovering 287 schizophrenia-associated loci.^28^ The cumulative effect of these variants can be summarized into polygenic risk scores (computed as the weighted sum of risk alleles under an additive model) that estimate the overall propensity for schizophrenia as indexed by common frequency variants (sczPRS).^29^ This major advance in genetic risk profiling, as well as the availability of data from large databases and consortia has spurred a recent explosion in studies exploring links between sczPRS and overt signatures of illness, including MRI brain measures commonly implicated in the pathophysiology of schizophrenia.

This systematic review aimed to comprehensively assess whether sczPRS relates to whole-brain and/or regional variation in commonly measured MRI-derived phenotypes of brain structure and function. To capture the breadth of brain phenotypes associated with schizophrenia, we included measures of T1-weighted structure, diffusion-weighted MRI (dMRI), and functional MRI (fMRI). We hypothesized that sczPRS would associate with features of brain structure and function, specifically in frontal, temporal, and insular cortices.

## Methods

### Literature Search Strategy

This systematic review was conducted in accordance with the Preferred Reporting Items for Systematic Reviews and Meta-Analysis(PRISMA systematic review protocol^30^ and was registered with PROSPERO (ID = CRD42021228902). Literature searches were run in MEDLINE, Embase, and PsycINFO on March 24, 2022, to search for eligible papers. Keywords included terms relevant to neuroimaging (eg, volume, surface area [SA]), genetics (polygenic risk score, PRS), and schizophrenia (eg, schizophrenia schizoaffective, and psychosis). The full search algorithm is available in supplementary material. Titles and abstracts were screened in Covidence platform post-de-duplication to exclude ineligible items. Subsequently, full texts were screened for inclusion based on criteria below.

### Manuscript Eligibility

We followed the PRISMA model^30^ when assessing studies for eligibility. Cohort, cross-sectional or longitudinal studies were included if they examined the association between sczPRS (based on genome-wide SNPs or SNPs comprising a specific gene set) and measures of brain structure and/or function obtained by MRI. Studies examining either community samples and/or schizophrenia cohorts were included.

Study inclusion criteria included the following: (1) peer-reviewed, (2) experimental (eg, not review articles), (3) published in the English language, and (4) sczPRS constructed from summary statistics reported in psychiatric genomics consortium 2 (PGC2) 2014^31^ or later. Quality control was conducted using the National Institutes of Health quality assessment tool (supplementary material). A manuscript with three or more “no”/“not reported” responses to these questions were excluded from the review.

### Polygenic Risk Profiles

Before describing the results, we note features of sczPRS that are important for interpreting reported results. First, the predictive efficacy of the sczPRS depends on the size of the GWAS discovery sample in which the SNP effect sizes were estimated. Since different discovery samples have different proportions of cases which impacts power, our approach to providing a fair comparison is given by knowledge of the number of genome-wide significant loci (n_GWS_) and by the effective sample size (Neff = 4 × (Ncase × Ncontrol)/(Ncase + Ncontrol)). Neff represents a study of equivalent power but with cases and controls equally represented. Nearly all studies reviewed here computed sczPRS based on the psychiatric genomics consortium wave 2 (PGC2) GWAS summary statistics (n_GWS_ = 108, Ncase = 36 989, and Ncontrol = 113 075, Neff = 111 487).^31^ Three studies^32–34^ constructed sczPRS based on Pardiñas et al 2018 (n_GWS_ = 145, Ncase = 11 260, Ncontrol = 24 542, Neff = 30 874).^35^ Only 2 studies^36,37^ constructed sczPRS based on the latest PGC3 GWAS (n_GWS_ = 287, Ncase = 76755, Ncontrol = 243 649, Neff = 233 471),^28^ another 2 used the Chinese-Ancestry GWAS (n_GWS_ = 113, Ncase = 7699 Ncontrol = 18 327 controls, Neff = 21 686) and trans-ancestry meta-analysis (Ncase = 43 175, Ncontrol = 65 166, Neff = 103 877).^38^

Second, the studies reviewed differ in the methods used to construct the PRS, which impacts the number of SNPs included and their weights. Nearly all studies used the simple clumping and *P*-value thresholding method (ie, selecting the most associated SNP in a locus with *P*-value less than a threshold [*P*_T_] and then only selecting additional SNPs to those already included if they are semi-independent based on a given linkage disequilibrium [LD] threshold). Many studies report results using a single *P*_T_ threshold, selected as the threshold suggested from prediction analyses provided in the discovery GWAS. This is an unbiased approach, compared to the studies which apply multiple thresholds in their own data and select the results from the *P*_T_ that maximizes the outcome in their own data, a form of overfitting. In both PGC2 and PGC3 papers, PRS analyses were presented and the *P*_T_ threshold that gave highest variance explained was *P*_T_ = .05, with variance explained on the liability scale in a held-out sample used for benchmarking was *R*^2^ = 8% for PGC2,^31^ and *R*^2^ = 10% for PGC3.^28^ In a study comparing PRS methods using PGC2 data, methods that modeled genetic architecture (eg, SBayesRm LDPred2, PRS-CS) explained a median of 12% variance in liability.^39,40^ Information on sczPRS construction is provided in supplementary tables to aid interpretability of the findings.

### Data Extraction

The following data were extracted from each manuscript: (1) sample characteristics (number of subjects, sex, and age), (2) imaging modality (T1 structural, dMRI, or fMRI), (3) MRI phenotype (cortical thickness, volume, SA, gyrification index, fractional anisotropy [FA], mean diffusivity [MD], axial diffusivity, radial diffusivity, functional network connectivity (FNC), and BOLD signals), (4) brain regions examined (whole-brain or regions/networks of interest), (5) GWAS utilized (eg, PGC2), (6) SNP shrinkage method to minimize LD (eg, clumping and pruning), (7) SNP *P*-value inclusion threshold/s (*P*_T_), (8) statistics characterizing associations between MRI phenotypes and sczPRS, and (9) covariates used in the association analyses. This review is qualitative as inter-study design differences, as well as lack of sufficient studies—the activation likelihood estimation meta-analysis model requires at least 17–20 experiments to yield robust analyses^41^—precluded a meta-analysis.

## Results

### Summary of Studies—Inclusion and Quality

**Fig. 1.**
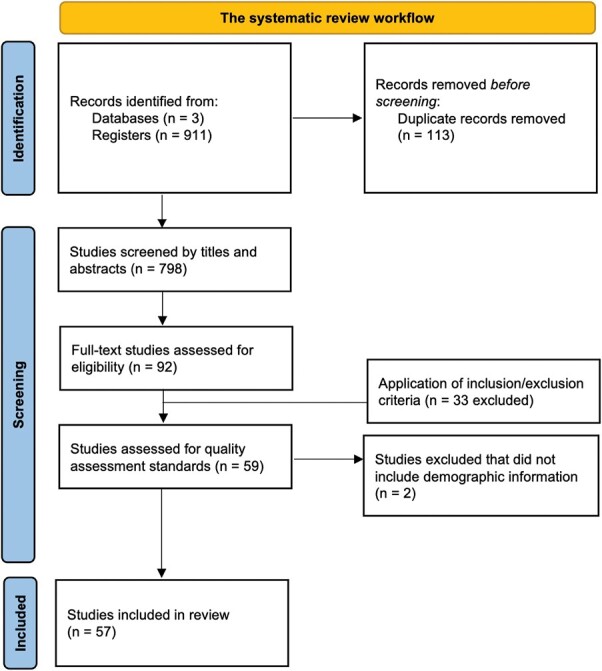
PRISMA diagram, a summary of methodology.

**Fig. 2.**
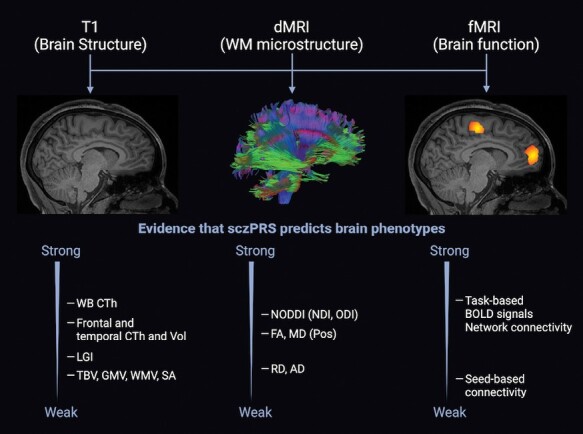
Qualitative summary of evidence for sczPRS associations with MRI-derived brain phenotypes. This summary draws from evidence based on the frequency and quality (eg, sample size) of reported significant effects, which are detailed in the Results. Reported associations were negative in direction unless stated otherwise. **Abbreviations**: AD, axial diffusivity; BOLD, blood-oxygen-level-dependent; CTh, cortical thickness; dMRI, diffusion magnetic resonance imaging; FA, fractional anisotropy; fMRI, functional magnetic resonance imaging; GMV, gray matter volume; LGI, local gyrification index; MD, mean diffusivity; NDI, neurite density index; NODDI, neurite orientation dispersion and density imaging; ODI, orientation density index; Pos, positive (denoting positive reported associations); RD, radial diffusivity; SA, surface area; TBV, total brain volume; WB, whole brain; WMV, white matter volume.

**Fig. 3.**
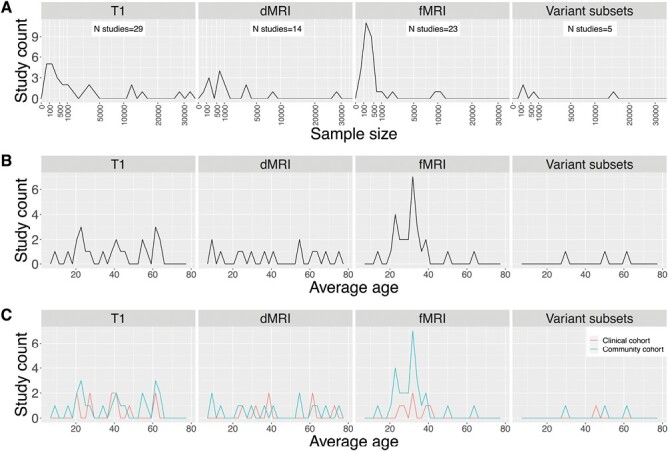
Summary of sample size, ages, and populations examined. **Panel A** shows the distribution of sample size across studies examining sczPRS associations. **Panel B** shows the age distribution across studies and **Panel C** shows the number of studies examining clinical and community populations.

The initial search generated 798 articles, 92 of which were assessed for eligibility using full-text articles. Out of these 92 studies, 59 met eligibility criteria. Of the remaining 59 studies, 57 satisfied quality assessment standards and were included for review (supplementary table 1). See figure 1 for the PRISMA diagram and supplementary figure 1A for the frequency of studies meeting each quality assessment criterion. The reviewed literature included studies that examined sczPRS in relation to brain phenotypes measured from T1 structural, dMRI, and fMRI. Several confounds, including demographic, MRI, and genetic-related factors were included in these analyses, which are summarized in supplementary figure 1B. An overall summary of sczPRS studies utilizing whole genome SNP sets is illustrated in figure 2. In addition, sample size distribution, age distribution, and cohorts examined are summarized in figure 3.

### Schizophrenia Polygenic Risk Associations With Structural T1-Weighted MRI Measures

A total of 29 studies examined associations between sczPRS and T1-weighted MRI measures that capture gross neuroanatomical features, including volume, thickness, SA, and the local Gyrification Index (LGI) (see supplementary table 2 for tabulated results).^33,36,37,42–66^

#### Volume.

Only one study investigated associations between sczPRS and total brain volume, which yielded no significant results.^45^ Regarding specific tissue contrasts, no studies out of 7 examining total gray matter volume observed significant associations with sczPRS.^36,43,45,46,60,63,65^ Likewise, 3 studies of total white matter volume did not reveal significant associations with sczPRS.^45,51,63^ Collectively, and consistent with the main conclusion drawn in a preliminary review,^67^ no evidence has emerged for sczPRS associations with whole-brain volumetric measures.

**Fig. 4.**
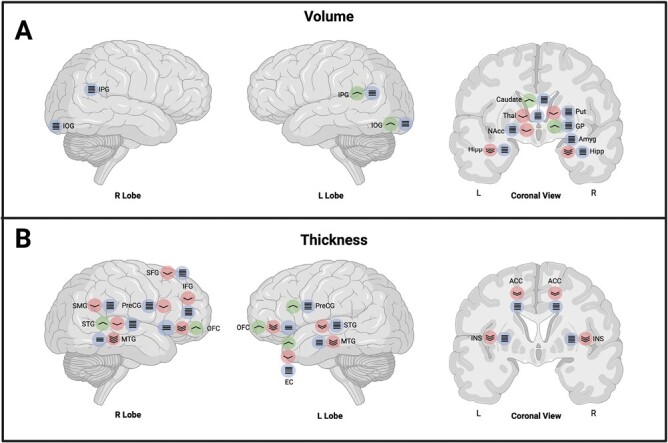
Regions showing significant associations with sczPRS. **Panel A** demonstrates regions with significant correlations between volume and sczPRS. **Panel B** highlights regional thicknesses that are significantly associated with sczPRS. Colors red, green, and blue code for negative, positive, and not significant associations respectively. Number of elements within each circle denotes the number of studies. **Abbreviations**: ACC, anterior cingulate cortex; Amyg, amygdala; EC, entorhinal cortex; GP, globus pallidus; Hipp, hippocampus; IFG, inferior frontal gyrus; INS, insula; IOG, inferior occipital gyrus; IPG, inferior parietal gyrus; MTG, medial temporal gyrus; NAcc, nucleus accumbens; OFC, orbitofrontal cortex; Pal, pallidum; PreCG, precentral gyrus; Put, putamen; SFG, superior frontal gyrus; SMG, supramarginal gyrus; STG, superior temporal gyrus; Thal, thalamus.

In terms of regional volumes (figure 4A), the only replicated finding was inverse sczPRS associations with hippocampal volume, which was reported in 3 independent studies.^52,54,56^ Notably, Harrisberger et al. (2016) identified this link separately in a community sample and individuals with a first-episode psychosis, suggesting that associations may not be driven by diagnostic status, or by variability in the clinical group.^52^ However, these significant findings fall in contrast to 6 other studies reporting no significant sczPRS association with hippocampal regions^33,36,48,50,53,63^ (see figure 4A), including one study utilizing UK Biobank data with a sample size of 27,632 and sczPRS constructed based on the latest PGC3 summary statistics.^36^

In terms of subcortical volumes, none of the 7 studies^33,44,45,48,51,53,61^ indicated significant associations with sczPRS. However, Sha et al. 2022^68^ reported significant associations between sczPRS and the asymmetry index of subcortical volumetric measures, which were negative in direction (ie, lower asymmetry relates to higher sczPRS) in the thalamus, putamen, hippocampus, and nucleus accumbens, and positive (higher asymmetry relates to higher sczPRS) in the globus pallidus and caudate.

In contrast to an intuitive negative correlation implying that higher sczPRS relates to lower brain volume, 2 studies reported positive associations between sczPRS with regional brain volumes. The first study highlights the left inferior parietal gyrus in young individuals with psychotic experiences, and the second study pinpoints regions across a subset of voxels within the right inferior occipital gyrus in a healthy aging population but not in a schizophrenia group,^62^ which was measured with a univariate voxel-based morphometry (VBM) analysis of gray matter volume. It is possible that VBM increased sensitivity to capture localized positive associations between sczPRS and brain volume; however, these preliminary VBM-derived results require replication.

#### Thickness.

Reduced cortical thickness (CTh), defined by the distance between the pial surface and the gray/white matter interface, is among the most robust MRI findings in schizophrenia, and is proposed to reflect reduced neurite density.^69^ Four^49,57,59,60^ out of nine studies^36,37,47,49,53,57,59,60,66^ identified significant negative correlations between sczPRS and whole-brain CTh (WB-CTh), with effect sizes (absolute Pearson’s r) ranging from 0.05 to 0.24 (*R*^2^ = 0.002–0.06).^59,60^ It is further noteworthy that while Neilson et al 2019^60^ (*N* = 2864) reported negative sczPRS—WB-CTh associations in a subset of samples comprising the UK Biobank, such findings were not replicated by Stauffer et al. 2021^36^ which examined a larger number of samples comprising the same dataset (*N* = 27 632). Lv et al. 2020^57^ and Neilson et al. 2017^59^ reported no effect of diagnosis on this relationship.

Nearly all (7 out of 8) studies examining measures of regional CTh reported significant associations, most of which were negative in direction (figure 4B), with Pearson’s r effect sizes ranging from 0.044 to 0.30 (*R*^2^ = 0.002–0.23). Studies involving community samples observed sczPRS correlations with CTh in the frontal lobe (bilateral orbitofrontal cortex,^42,44,68^ right inferior frontal gyrus and right precentral gyrus),^44^ temporal lobes (medial temporal lobe,^42,44^ and the middle^44^ and superior temporal cortices),^50,68^ and bilateral insula.^43,60^ These regional findings have been observed separately in community cohorts^44,50,60,68^ and in a mixed cohort of healthy controls and individuals with schizophrenia.^42,43^ In contrast, an effect of diagnosis was evident in one study that observed a negative sczPRS-CTh association in the left temporal lobe among individuals with schizophrenia but not in a control group.^59^ Furthermore, in a cohort of individuals diagnosed with a major depressive disorder, a *positive* association was detected between sczPRS and CTh of the rostral anterior corpus callosum (rACC), superior temporal gyrus, and entorhinal cortex, the opposite of that observed in a healthy control group.^50^ A possible interpretation of such results is the involvement of different biological pathways in major depressive disorder despite the presence of high sczPRS, but this hypothesis needs further evaluation.

Despite the above studies reporting significant sczPRS associations with region-specific variation in CTh, the findings are mixed. Indeed, for each study reporting a significant regional sczPRS association, more studies reported no significant effect (figure 4B). Of note, no significant CTh-sczPRS associations were found in Stauffer et al. 2021, which utilized UK Biobank data (N = 27 632).^36^

As opposed to raw thickness values, one study reported sczPRS associations with the asymmetry index of regional thickness, with negative associations (lower asymmetry relates to higher sczPRS) localized to the parietal lobe and rACC and positive associations (higher asymmetry relates to higher sczPRS) localized to frontal and temporal regions.^68^ Notably, these findings were obtained using summary statistics from PGC2 with analyses using inferred effect sizes from Bayesian shrinkage.

#### Surface area.

There is less evidence for SA alterations in schizophrenia relative to brain volume and thickness measures.^70–72^ Perhaps consistently then, none out of 4 studies examining SA reported significant sczPRS associations with whole-brain or regional measures in community samples.^37,50,53,60^ In contrast, one study examining associations between sczPRS and the asymmetry index of regional SA measures reported several associations (both positive and negative) with Pearson’s r ranging from 0.02 to 0.39 (*R*^2^ = 0.0004 to 0.15).^68^

#### Local Gyrification Index.

Cortical folding is commonly summarized into a local Gyrification Index (LGI), reflecting the ratio of outer folded surface length to the outer surface length excluding sulci. Lower LGI is frequently reported in diverse schizophrenia populations.^73^ Two out of four studies^36,47,55,58^ examining LGI reported negative associations with sczPRS with LD-pruned PGC2-derived SNPs, which were localized to the left medial orbitofrontal cortex in individuals with persistent psychotic experiences,^47^ and to the inferior parietal lobes in healthy individuals.^55^ In contrast, positive sczPRS- LGI associations were observed in frontal regions among individuals with high familial risk of schizophrenia and with established schizophrenia-spectrum disorders.^58^ However, these results were not replicated by Stauffer et al, which utilized an extended dataset of UK Biobank with PGC3-derived sczPRSs.^36^ Thus, LGI-sczPRS associations may vary across specific populations, and/or as a function of PRS construction methods.

### Schizophrenia Polygenic Risk Associations With dMRI Measures in White Matter Tissue

A total of 14 studies have examined sczPRS associations with dMRI metrics (see supplementary table 3),^32,36,43,45,50,57,63,66,74–79^ which summarize the diffusion of water molecules in the brain and is sensitive to microstructural changes in white matter architecture, including in the myelin sheath, microtubules, and neurofilaments.^80^

#### Fractional Anisotropy.

FA is the most commonly used scalar measure to summarize dMRI signals, which is derived from diffusion tensor imaging (DTI)—the simplest and earliest model considered in dMRI.^81–83^ Despite ubiquitous evidence for reduced FA in schizophrenia, only three^36,77,78^ out of fourteen studies^32,36,43,45,50,57,63,66,74–78,84^ observed a main association between sczPRS and white matter FA, with small reported effect sizes of ~0.01. A significant effect was observed recently by Su et al. 2022, which constructed sczPRS based on SNPs identified in a Chinese-ancestry population.^77^ They reported negative sczPRS-FA associations in the genu and body of the corpus callosum and in the right anterior and superior corona radiata in individuals with a first episode of schizophrenia (FES), but not in controls.^77^ Three other studies included schizophrenia cohorts,^57,74,76^ which did not detect significant FA-sczPRS correlations; however, the clinical group was either small (SCZ *n* = 28 in Alloza et al. 2017^74^ and SCZ *n* = 21 in Simões et al. 2020)^76^ and/or characterized by older individuals with prolonged illness.^57,74^ As such, sczPRS-FA associations may manifest around the typical age of psychosis onset (late adolescence and early twenties), specifically in a FES population. Most studies in healthy or community populations,^32,36,43,57,66,74,75^ or in other psychiatric populations, including MDD^50^ and bipolar disorder^76,84^ reported no such association between sczPRS and FA.

Of 2 additional studies reporting significant sczPRS-FA associations, one observed a positive correlation (Pearson’s *r* = 0.011, *P* = .03) among healthy children.^78^ The other represents perhaps the strongest evidence for sczPRS-white matter FA associations to date, which utilized the largest sample (a community sample drawn from the UK Biobank).^36^ This study observed significant negative correlations between sczPRS and FA in several major white matter tracts, which was most pronounced in the forceps minor. Despite this key finding, evidence for sczPRS associations with white matter FA is collectively inconclusive; with limited evidence constrained to a large community dataset and younger healthy and FES cohorts comprised of small sample sizes.

#### Other Diffusivity Measures.

Only a handful of studies (*n* = 7) have examined sczPRS in relation to other dMRI measures, with 3 reporting significant associations. In a longitudinal study of healthy older individuals (mean age at baseline was 73, with follow-up scans after 3 years), sczPRS was positively correlated to longitudinal MD increases in the splenium,^32^ suggests that *changes* in MD—a measure of overall diffusivity^85^—may be more sensitively linked to variability sczPRS, relative to cross-sectionally-measured and alternative DTI measures. More nuanced relationships between sczPRS and other DTI measures have also been described. Specifically, moderation analysis revealed that higher sczPRS in the presence of high cortisol levels predicted higher axial diffusivity and radial diffusivity in young adolescents.^45^ In a large cross-sectional study, Stauffer et al. 2021 (mentioned above) reported significant positive associations between MD and sczPRS,^36^ in 11 out of 15 white matter tracts. Furthermore, they reported negative associations between sczPRS and more advanced dMRI measures with increased biological specificity, namely, the neurite density index and orientation density index, in a community population sample (UK Biobank), with Pearson’s r ranging from 0.0165 to 0.0238 (*R*2 = 0.00002 to 0.0008).^36^ Therefore, relationships between measures of white matter microstructure and sczPRS appear characterized by nuances in time, biology, and sample size. Alternative study designs (eg, longitudinal) and more advanced dMRI measures have provided greater clarity and specificity in differentiating biologically distinct microstructural associations with sczPRS.

### Schizophrenia Polygenic Risk Associations With fMRI Measures

A total of 23 studies have examined sczPRS associations with brain functional architecture (see supplementary table 4).^42,53,54,56,77,86–103^ These can be broadly categorized into studies of (1) resting-state functional MRI (rfMRI), measuring local or synchronous inter-regional BOLD fluctuations at rest, and (2) task-based fMRI (tbfMRI), which identify brain regions that are functionally involved in task performance.

#### Resting-State fMRI.

Reduced functional connectivity, as observed by rsfMRI, is widely reported in schizophrenia,^4,104^ and has been analyzed with regard to sczPRS associations in 9 studies.^42,56,77,86,87,89,99,101,102^ Of these, 7 investigated FNC, with 4 reporting significant negative associations across community samples,^87^ healthy individuals,^56,101^ and in schizophrenia patient populations.^56,77,101^ Notably, these associations were found with different functional measurements, which preclude direct comparison. Specifically, negative associations were observed between sczPRS and local BOLD signals in the visual cortex^86^ and with BOLD signal coupling within frontal and between frontal and temporal regions, based on functional disruptions implicated in a FES group.^77^ In a different approach, Liu et al. 2020 identified inverse sczPRS associations with BOLD signal coupling between hippocampal seed regions and all remaining gray matter voxels.^56^ In addition to studies of functional coupling, 3 of the 7 studies producing significant associations applied a connectome-based approach. These have identified inverse associations between sczPRS with (1) mean connectivity across principal network components involving visual, default-mode, and frontoparietal systems,^87^ (2) the first independent component of dynamic functional connectivity obtained by independent component analysis,^101^ and (3) with greater group (schizophrenia-control) differences in FNC.^89^ These studies collectively implicate genetic liability for schizophrenia in reduced functional connectivity across diverse brain networks.^87^

#### Task-Based fMRI.

A total of 16 studies have examined functional measures during a task paradigm. Working memory constitutes the most studied fMRI task and nearly all of these (8 out of 9), including studies of BOLD signals, FNC, and effective connectivity reported significant effects.^87,88,91,93–96,100^ These included studies of community samples,^87,88,91,94–96,100^ unaffected healthy relatives of individuals with schizophrenia,^93^ and schizophrenia populations.^95^ Significant negative associations between sczPRS and working-memory-based functional measures were frequently localized to frontal regions with Pearson’s r effect sizes ranging from −0.28 to −0.15 (*P* < .05)^94,95,100^; however, inconsistencies appeared at finer resolutions, with no 2 studies reporting effects in the same specific areas within the frontal lobe.

Three tbfMRI studies utilized emotion perception tasks, which produced mixed results. Specifically, Erk et al. 2017^93^ did not detect associations using a face-matching task that measured implicit processing of negative emotions,^105^ Dzafic et al. 2018^92^ observed a positive sczPRS correlation with dorsolateral prefrontal cortex (dlPFC) activity during threat-evoking emotion perception task. Cao et al. 2020^87^ reported negative associations between sczPRS and whole-brain FNC during an emotional face-processing task.^106^ As such, mixed findings may relate to differences in the specific emotion perception task utilized, and different neural circuitry involved in threat and face processing.

Very few studies have examined functional measures during other tasks and conclusions are limited due to a lack of overlap between the tasks examined. In general, these studies have reported negative and/or positive associations between sczPRS and functional measures during learning tasks,^53,97^ reward-processing or gambling tasks,^98,103^ motor, social cognition, relational processing, and language processing,^87^ theory of mind,^93^ and facial expression tasks.^54^

### Brain Associations With sczPRS Limited to Variant Subsets

Several studies (see supplementary table 5) have characterized brain morphological associations with cumulative genetic risk computed from subsets of schizophrenia risk variants based on gene sets compiled from different sources.^33,34,65,107,108^ For brevity, these results have been relegated to supplementary material.

### Impact of Sample Size on sczPRS Associations With MRI Phenotypes

**Fig. 5.**
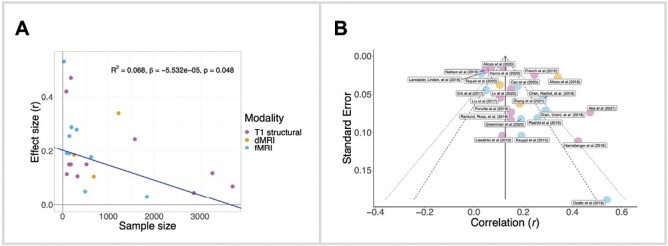
Effect size analysis in studies reporting significant sczPRS-phenotype associations. **Panel A** shows the association between sample size and effect size (reported in Pearson’s r). Studies across T1 structural, dMRI, and fMRI modalities were included if they reported a significant association between any brain phenotype and sczPRS. This resulted in 23 out of 57 studies and 23 datapoints, each reflecting the average of effect sizes for significant association observed in the relevant studies. The correlation controls for imaging modality. The excluded studies did not indicate any significant sczPRS and phenotype associations (*n* = 12), did not report standardized effect sizes (15), or reported on gene-set-constrained sczPRS (*n* = 6). One additional study (Stauffer et al. 2021) was excluded which was an outlier in sample size (*n* > 27 000 participants). **Panel B** displays a funnel plot of the same studies, with the correlation in Pearson’s r on the x-axis and the standard error derived from Pearson’s r and sample size on the y-axis. The solid vertical line represents the sample size-weighted mean correlation coefficient calculated across all studies (r̅) and the dashed and dotted diagonal lines represent the limits of the 95% and 99% confidence regions, respectively.

figure 5A displays the mean absolute effect size for each study reporting significant sczPRS-phenotype associations. As shown, effect size was negatively correlated to sample size using linear regression when using linear regression (*R*^2^ = 0.068, *β* = −5.5E-05, *P*-value = .048, *n* = 1000 permutations) after controlling for imaging modality (T1, dMRI, and fMRI, using a binary matrix for study membership). The correlation suggests that effect size decreases with greater sample size. Publication bias was assessed using a funnel diagram^109^ (figure 5B), which plots the correlation coefficient against the standard error for each respective study. An unbiased sample would show datapoints that fall within the limits of the 95% and 99% confidence regions and are symmetrically distributed around the population mean effect size (the solid vertical line). While most studies fall within the confidence regions, a few studies do not and thus, deviate unexpectedly from the population norm. These funnel plot results may indicate the presence of publication bias.

## Discussion

Two previous systematic reviews did not yield substantive evidence for associations between sczPRS and brain structure.^67,110^ Here, we expand on this work by reviewing a deluge of recent studies that have examined associations with sczPRS and a range of MRI measures of brain structure, microstructure, and function. Across ten MRI measures examined, whole-brain cortical thickness and resting-state functional connectivity measures revealed consistent evidence of association with polygenic risk for schizophrenia. In terms of other measures, including regional brain structural and dMRI measures, the findings are mixed, which may relate to differences in sample size/composition (eg, genetic ancestries), population characteristics (community vs schizophrenia), brain regions examined, and the GWAS used to construct sczPRS. These design differences precluded meta-analysis; however, allowed us to infer the following qualitative conclusions.

In terms of brain structure, whole-brain CTh showed moderate evidence for association with sczPRS, characterized by negative correlations. However, Stauffer et al. 2021^36^ reported no significant CTh-sczPRS associations in a community sample drawn from the UK Biobank with N = 27 632, which was the largest examination of WB-CTh with sczPRS to date. Lack of replicability by Stauffer et al. 2021 thus weakens the evidence base for sczPRS contributions to WB-CTh. At the brain regional level, significant results were often localized to frontal and temporal brain regions; however, the findings were mixed and not reproduced in the largest study using sczPRS based on the latest PGC3 GWAS.^36^ Despite the discrepancies, significant regional associations were reported across community and schizophrenia samples and different developmental epochs, supporting the reproducibility of associations across different populations. Reduced frontal-temporal thickness is among the most robust MRI findings in schizophrenia,^69,111–113^ which may predate psychosis onset^114^ and relate to both positive and negative symptoms. Furthermore, twin studies have shown reduced temporal thickness in unaffected first-degree relatives with schizophrenia that are intermediate to differences between patients and healthy individuals.^115^ These previous results together with findings from this review imply developmental sources of reduced cortical thickness in schizophrenia, particularly at a coarse (whole-brain) level.

While the review did not provide evidence for sczPRS associations with global volumetric measures, associations were observed between sczPRS and regional brain volumes, including the hippocampus in studies examining genome-wide and gene-set-constrained sczPRS. Reduced hippocampal volumes as well as associated deficits in working memory are frequently reported in schizophrenia.^116,117^ Some postmortem evidence implicates altered cell proliferation and neuronal differentiation in the hippocampi of schizophrenia-affected brains,^118^ which may point to altered hippocampal neurogenesis as a potential mechanism linking sczPRS to reduced hippocampal volume. This hypothesis is supported by enrichment of neurogenesis pathways in sczPRS,^119^ as well as in the genetic architecture of hippocampal volume, the latter of which was identified in a large-scale GWAS (*N* = 13 163) of hippocampal volume.^119^ Our review of gene set studies (see supplementary material) also suggested that polygenic risk in complement-enriched genes is linked to smaller volumes within hippocampal regions.^108^

In terms of brain function, task-based fMRI studies utilizing working memory paradigms revealed consistent evidence for negative associations with sczPRS, which mainly implicated frontal^53,95,96^ and temporal regions.^54,94,100^ However, these effects may not be constrained to state (or paradigm)-dependent connectivity, given that whole-brain and connectome-based studies identified sczPRS associations with widespread systems. For example, Cao et al. 2020 observed sczPRS associations with functional activity evoked by 8 independent paradigms, implicating widespread functional connectivity of brain systems.^87^ This result is consistent with cumulative evidence that connectivity is highly heritable^120^ and that schizophrenia represents a disorder of brain dysconnectivity (ie, disrupted communication within and between multiple brain regions).^121^

Insofar as most studies did not identify associations between sczPRS and white matter microstructure and that FA associations were observed in a FES group and not in controls,^77^ it is possible that abnormalities in these measures exhibited by individuals with schizophrenia are not entirely rooted in development but rather reflect secondary pathology driven by primary biological events or environmental/illness-related exposures. This hypothesis is supported by one of the reviewed studies that observed indirect impacts of sczPRS on white matter FA and MD, which was mediated by cortisol-indexed stress levels.^45^ Alternatively, it is possible that sample sizes were too small in most studies or that dMRI measures computed from the conventional DTI model, including FA, may be overly coarse, limiting the sensitivity of genetic risk to these measures. Indeed, Stauffer et al. examined advanced dMRI metrics constructed from neurite orientation dispersion and density imaging and observed more frequent relationships between sczPRS and the neurite density, compared to FA.^36^ As such, it is possible that more biologically specific white matter markers increase mechanistic links between genotype and phenotype.

It is noteworthy that effect sizes across all brain measures were generally small, as highlighted in figure 5A (less than 2% of variance explained). Thus, while evidence emerged in support of significant sczPRS associations with some brain measures, cumulative genetic risk for schizophrenia, at least as defined by sczPRS, is not a potent predictor of variability in any brain measure considered. Weak associations may call to question the biological relevance of sczPRS-brain associations and suggest that alternative genetic or environmental factors drive variability in brain measures commonly implicated in schizophrenia pathophysiology, as well as possible non-additive gene-by-environment effects. Notably, sczPRS associations with clinical symptoms and severity, as well as with environmental exposures^122–125^ have yielded higher effect sizes than those observed here between sczPRS and brain phenotypes. For example, significant correlations have been observed between sczPRS with illness severity (*r* = −0.28, *R*^2^ = 0.08)^122^ and cognitive performance (*r* = −0.11 to −0.08,^123^*r* = −0.65 to 0.020),^124^ although these findings are mixed, with some reporting no significant link.^126,127^ Collectively, the small effect sizes observed here, together with previous work examining sczPRS associations with clinical phenotypes, suggest that genetic risk loci for schizophrenia relate, albeit weakly, to broad-ranging clinical and brain phenotypes implicated in schizophrenia.

In addition to small effect sizes, we also observed a negative association between effect size and sample size, which may suggest an overestimation of effect sizes in studies with smaller sample sizes. This pattern is consistent with the previous literature on publication bias in psychological science and brain imaging literature.^128,129^ We evaluated this possibility using a graphical funnel plot method, which suggested the presence of publication bias (figure 5B). This finding is consistent with the “winner’s curse,” ie, published studies are over-representative of repeated samplings in various populations. The potential for publication bias obscures conclusions regarding sczPRS associations with brain phenotypes. This challenge is highlighted by the sczPRS-CTh findings: While the majority of studies examining sczPRS associations with whole-brain CTh reported a significant relationship, the largest study to date (Stauffer et al. 2021) did not, which tempered our conclusions regarding genetic risk contributions to WB-CTh. Furthermore, if we exclude studies falling outside the confidence intervals in figure 5B (ie, studies with higher/lower expected effect size), only two^57,59^ out of seven studies^36,37,47,53,57,59,66^ would show a significant sczPRS-WB-CTh association, albeit with a net effect size consistent with the main conclusions (as both studies with higher and lower effect sizes would be removed). Given these complexities and potential effects of publication bias, it is imperative to reexamine such relationships to ensure replicability and to minimize effect size inflations observed in smaller studies.^130^ Smaller studies may nonetheless complement large-scale studies and enable validations of findings and testing of their generalizability in other populations and cohorts, particularly in light of possible selection bias in large datasets, such as the UK Biobank.^131^

### Limitations

First, we could only address sczPRS-brain phenotype associations with studies of European ancestry, which is a reflection of the published brain imaging studies. Furthermore, the most frequently used GWAS to calculate sczPRS (PGC2) was based on samples of mainly (94%) European descent. Recent GWASs, including the new PGC3, have since identified new genome-wide significant loci based on samples with mixed European, African, African American, East-Asian, and Latino ancestries; however, only 2 studies^36,37^ included in this review utilized these latest summary statistics (due to the recency of publication). Second, our findings cannot account for recent reports of substantial heterogeneity observed by normative modeling studies of brain structure in schizophrenia.^57,132^ It is possible that the strength of sczPRS-phenotype associations differ across schizophrenia populations and are influenced by various environmental and illness-related factors. This heterogeneity may also underlie discrepant findings at the brain regional level in measures of brain structure. Third, we did not account for variation in genotype quality control procedures (eg, missingness, relatedness, and minor allele frequencies) but PRS are very robust to these measures as many combinations of SNPs provide equivalent PRS. Fourth, a small fraction of studies did not report on methods to enforce control over multiple comparisons across brain regional analyses (refer to supplementary tables for details), which limited reliance on these findings in our analysis of the extant literature. Therefore, these few studies did not alter any major conclusions in this systematic review. Fifth, there was a lack of independence in terms of samples between all the studies considered in this systematic review. In particular, Alnæs et al. 2019,^44^ Neilson et al. 2019,^60^ Sha et al 2021,^68^ and Stauffer et al. 2021^36^ utilized the UK Biobank dataset, and therefore cannot be considered as fully independent. Despite sample overlap, different conclusions were drawn: Neilson et al. reported negative sczPRS-whole-brain CTh correlations, in contrast to nonsignificant results in Stauffer et al. Moreover, Alnæs et al. and Neilson et al. reported regional sczPRS-CTh associations in inconsistent regions. While the discrepant results may be due to the GWAS used to calculate polygenic risk scores (PGC3 in Stauffer et al. 2021^36^ and PGC2 in Neilson et 2019),^60^ the inconsistent findings in studies using overlapping samples reduce confidence in the reliability of sczPRS-CTh associations.

Our review finds moderate support for sczPRS associations with whole-brain cortical thickness, and brain-wide functional connectivity measures, as well as moderate/mixed evidence for sczPRS associations with gray matter thickness and volumes of specific frontal and temporal regions. Less consistent evidence emerged for sczPRS links with whole-brain volume and SA, as well as measures of white matter microstructure. These findings were broadly consistent across community and patient samples. Future studies could employ advanced MRI techniques to account for specific biological compartments and explore indirect pathways and environmental factors that mediate genetic risk associations with brain phenotypes associated with schizophrenia.

## Supplementary Material

sbad087_suppl_Supplementary_Material

sbad087_suppl_Supplementary_Tables
