# Supplementary material for: Linking Polygenic Risk of Schizophrenia to Variation in Magnetic Resonance Imaging Brain Measures: A Comprehensive Systematic Review
Source: Schizophr Bull. 2023 Jun 24;50(1):32–46. doi: 10.1093/schbul/sbad087 (PMC10754175; doi:10.1093/schbul/sbad087)
Supplement: sbad087_suppl_Supplementary_Material [file sbad087_suppl_supplementary_material.docx]

# Supplementary Material

Table of Contents

[Supplementary Material 1](#_Toc136422058)

[Supplementary Methods 1](#_Toc136422059)

[Search used to identify relevant articles 1](#_Toc136422060)

[Quality assessment 2](#_Toc136422061)

[Supplementary Results 3](#_Toc136422062)

[Brain associations with sczPRS limited to variant subsets 3](#_Toc136422063)

[References 4](#_Toc136422064)

## Supplementary Methods

### Search used to identify relevant articles

*(schizophreni* OR SCZ OR psychosis OR psychoses OR psychotic OR psychotic- like OR schizoaffective OR schizotyp* OR catatoni*).ti,ab.*

*AND*

*(PRS OR polygenic risk score OR polygenic).af*

*AND*

*(neuroimag* OR brain imaging OR magnetic resonance imaging OR MRI OR structural OR T1 OR diffusion OR diffusion-weighted imaging OR DWI OR functional magnetic resonance imaging OR functional MRI OR fMRI OR brain volume OR thickness OR cortex OR cortical OR surface area OR grey matter OR GMV OR white matter OR microstructure OR white matter microstructure OR WMV OR fractional anisotropy OR FA OR brain network OR streamline* OR effective connectivity OR connectivity OR connectom*).ti,ab.*

**Options applied to search:** Non-peer reviewed studies, non-human studies, and articles not published in the English language were excluded while searching the databases.

### Quality assessment

Quality control was conducted using the NIH quality assessment tools. A total of 11 questions were addressed for each study. Studies were excluded if they did not contain demographic information (sex, ethnicity, educational level, IQ), genotyping protocol, MRI acquisition parameters, and clearly defined and consistent outcome variables (i.e., the same outcome variables were applied across populations). The questions considered for quality control assessment are shown in Supplementary Table 1. In addition, results from the quality control assessment are summarized in Supplementary Figure 1.


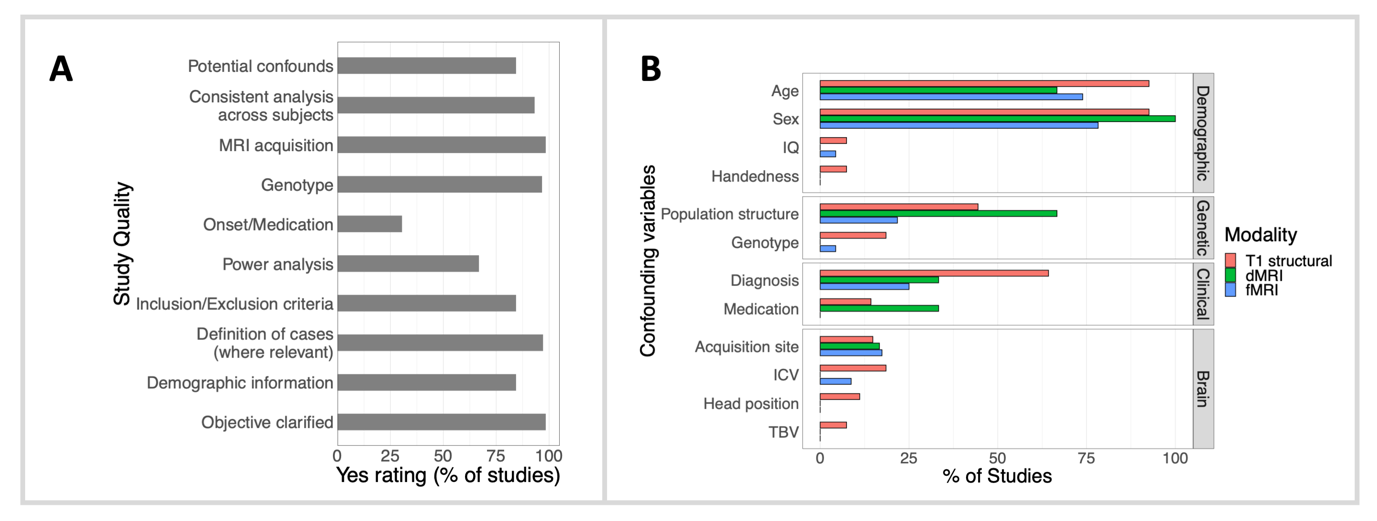


**Supplementary Figure 1. Quality assessment criteria and confound inclusion.** **Panel A** shows the frequency of studies with a “yes” response to each quality assessment criteria. **Panel B** shows confounding variable occurrence across studies in terms of association analyses between brain phenotypes and sczPRS. Studies examining gene-sets (as opposed to genome-wide sczPRS) are not included in Panel B. **Abbreviations:** dMRI = diffusion magnetic resonance imaging, fMRI = functional magnetic resonance imaging, ICV = intracranial volume, IQ = intelligence quotient, TBV = total brain volume.

## Supplementary Results

### Brain associations with sczPRS limited to variant subsets

The 108 risk loci identified in the 2014 GWAS^31^ have been categorized into six functional groups: synaptic function and plasticity (9 genes); neurodevelopment (7 genes); neuronal ion channels (5 genes); calcium signaling (7 genes); therapeutic targets (2 genes); and glutamatergic neurotransmission (6 genes).^31^ Embedding genetic risk variants within each gene set did not yield conclusive evidence of functional-specific sczPRS associations with brain volume.^59^ Among the aforementioned gene sets, only polygenic risk for synaptic transmission (the weighted sum of KCTD13, NLGN4X, IGSF9B, CNTN4, MEF2C, PTN, CNKSR2, PAK6, and SNAP91 variants) showed nominal effects on reduced parahippocampal volume; however, this analysis did not control for genome-wide polygenic risk for schizophrenia or perform permutations to control for background genetic signal.^59^

In another approach, specific biological pathways have been investigated. For example, using sczPRS limited to dopaminergic transmission genes higher scores were associated with disrupted connectivity in working memory.^102^ In addition, inflammation-linked sczPRSs have been investigated in sczPRS-MRI association studies, given known roles of the major histocompatibility complex (MHC) region in schizophrenia risk, arising in part from alleles of the complement component 4 (C4) genes.^103^ Holland et al. recently showed an effect of sczPRS limited to 90 complement-enriched immunity genes on significantly smaller volumes within hippocampal regions, which was observed in a combined group of healthy controls and individuals with schizophrenia, as well as just in healthy controls.^104^ A related study examined sczPRS limited to microglial cell-linked genes on gray matter volume in a mixed group of healthy individuals and patients with schizophrenia though failed to detect significant associations.^105^ As recently shown, a relationship between microglial-linked sczPRS may only be evident in a subset of individuals with schizophrenia.^106^ Thus, patient heterogeneity may obscure relationships with cell type-specific PRS identified in community samples and schizophrenia cohorts.

In a final approach, Grama et al. 2020 examined brain associations with sczPRS computed on gene sets enriched for genetic association with schizophrenia—abnormal behavior, abnormal long-term potentiation, abnormal nervous system electrophysiology, FMRP targets, 5HT2C channels, CaV2 channels, and ‘loss-of-function intolerant genes’.^40^ Only an abnormal behavior-PRS, significantly associated with brain volume, was characterized by a negative correlation with left and right thalamic volumes in a large community sample drawn from the UK Biobank.^40^ Abnormal behavior, characterized by atypical actions or reactions to stimuli, is a common symptom of schizophrenia whose gene set is enriched for neurological learning pathways.^107^

## References

**1.** Consortium SWGotPG. Biological insights from 108 schizophrenia-associated genetic loci. *Nature* 2014;511(7510):421-427.

**2.** Alloza C, Cox SR, Blesa Cábez M, et al. Polygenic risk score for schizophrenia and structural brain connectivity in older age: A longitudinal connectome and tractography study. *NeuroImage* 2018/12/01/ 2018;183:884-896.

**3.** Grama S, Willcocks I, Hubert JJ, et al. Polygenic risk for schizophrenia and subcortical brain anatomy in the UK Biobank cohort. *Translational Psychiatry* 2020/09/09 2020;10(1):309.

**4.** Corley E, Holleran L, Fahey L, Corvin A, Morris DW, Donohoe G. Microglial-expressed genetic risk variants, cognitive function and brain volume in patients with schizophrenia and healthy controls. *Translational Psychiatry* 2021/09/23 2021;11(1):490.

**5.** Pardiñas AF, Holmans P, Pocklington AJ, et al. Common schizophrenia alleles are enriched in mutation-intolerant genes and in regions under strong background selection. *Nature Genetics* 2018/03/01 2018;50(3):381-389.

**6.** Stauffer E-M, Bethlehem RAI, Warrier V, Murray GK, Romero-Garcia R, Seidlitz J, Bullmore ET. Grey and white matter microstructure is associated with polygenic risk for schizophrenia. *Molecular Psychiatry* 2021/12/01 2021;26(12):7709-7718.

**7.** Karcher NR, Paul SE, Johnson EC, et al. Psychotic-like Experiences and Polygenic Liability in the Adolescent Brain Cognitive Development Study. *Biological Psychiatry: Cognitive Neuroscience and Neuroimaging* 2022/01/01/ 2022;7(1):45-55.

**8.** Trubetskoy V, Pardiñas AF, Qi T, et al. Mapping genomic loci implicates genes and synaptic biology in schizophrenia. *Nature* 2022/04/08 2022.

**9.** Li Z, Chen J, Yu H, et al. Genome-wide association analysis identifies 30 new susceptibility loci for schizophrenia. *Nature genetics* 2017;49(11):1576.

**10.** Privé F, Arbel J, Vilhjálmsson BJ. LDpred2: better, faster, stronger. *Bioinformatics* 2020;36(22-23):5424-5431.

**11.** Ge T, Chen C-Y, Ni Y, Feng Y-CA, Smoller JW. Polygenic prediction via Bayesian regression and continuous shrinkage priors. *Nature Communications* 2019/04/16 2019;10(1):1776.
